# Uncertainty in determining the optimal test duration for maximal rate of lactate accumulation during all‐out sprint cycle ergometry

**DOI:** 10.1007/s00421-024-05506-2

**Published:** 2024-05-29

**Authors:** Woo-Hwi Yang, Benedikt Johannes Meixner, Billy Sperlich

**Affiliations:** 1 Graduate School of Sports Medicine, CHA University, Gyeonggi-do, Republic of Korea; 2 Department of Sport Science and Sport, Friedrich-Alexander-Universität Erlangen-Nürnberg, Gebbertstraße 123b, 91058 Erlangen, Germany; 3 Iq-Move PG Lochmann & Fraunberger, Gebbertstraße 123b, 91058 Erlangen, Germany; 4 Integrative and Experimental Exercise Science & Training, Institute of Sport Sciences, University of Wuerzburg, Judenbuehlweg 11, 97082 Würzburg, Germany

**To the Editor:**

We acknowledge the contribution of Langley et al. to the growing body of research on the validity of maximal accumulation of blood lactate (νLa_max_) after sprint cycling and associated metabolic processes. Their methodology, which utilizes varying test durations, offers additional perspectives on this developing subject. Nonetheless, we must emphasize three significant issues concerning the type of testing that future research in this domain should also consider:

## Ergometer use for νLa_max_ testing

A prerequisite for νLa_max_ testing is achieving maximum sprint power output, defined as the highest angular force per unit time generated by the cyclist’s muscles at maximum revolutions per minute. Although different methodologies exist, νLa_max_ testing is usually conducted in isokinetic mode set to 120–130 RPM on an ergometer designed for this mode of operation.

In the study by Langley et al. νLa_max_ testing was conducted on a WattBike ergometer functioning with linear resistances and does not support isokinetic mode. This limitation affects the test’s validity for *maximal* power output assessments, as both force and velocity constraints impact power outputs under these conditions. Notably, a documented decrease in mean power outputs over 15 s and 30 s intervals (see Fig. 6) had to necessitate a significant reduction in mean cadence. We argue that this reduction in cadence does not create an optimal circumstance for maximal glycolytic fiber activation (Driss and Vandewalle 2013). This factor could also contribute to the observed increase in oxidative energy contribution during longer durations (15 s and 30 s) compared to shorter 10-s intervals.

While the WattBike's force sensor samples at 100 Hz, the sampling rate of angular velocity is contingent upon the angular velocity itself. Prior validation studies have noted that the Wattbike assesses angular velocity—a critical component in the power calculation formula [P = (F * l/t)]—twice per pedal revolution (Hopker et al. 2010). Due to the relatively low pedal rates during acceleration, the WattBike might not suit the evaluation of traditional alactic time metrics in sprint cycling.

## Selection of study participants

The study’s participant composition, comprising 11 team sport players, 2 runners, and only 2 triathletes who regularly cycle, is problematic for several reasons: (i) the predominance of inexperienced cycling among the participants limits the generalizability of the findings to seasoned cyclists; (ii) the lack of routine sprint cycling experience for most participants means that, despite a familiarization session, their untrained cycling techniques could inhibit achieving the high pedaling rates necessary for substantial power outputs in this context; (iii) conversely, cyclists with habitual engagement in both regular and sprint cycling are likely capable of maintaining maximal glycolysis for longer durations.

Furthermore, the WattBike accommodates both strap and cleat cycling, but it remains uncertain if participants wore regular sports shoes or cycling-specific cleated shoes. We consider the employment of cycling shoes and an appropriate pedal system crucial for all-out cycling.

Lastly, we could only identify 14 participants in Figs. 5C, 5F, 6C and 6F for the 30-s test duration, despite 15 being reported in the methodology. Given the relatively small sample size, the absence of data from one participant could potentially lead to different conclusions.

## Calculation method

Although frequently considered negligible, we recognize the significance of measuring oxidative contributions to sprinting (Yang et al. 2023). While the authors utilize the energy system contribution calculations by Yang et al. (2023), we question their choice to disregard Yang et al.’s proposed pure νLa_max_ calculation method, which also accounts for oxidative energy-contributed time. This particular calculation method could help address discrepancies between test durations by considering the increase in oxidative metabolism over extended periods.

Within their discussion Langley et al. state that “Contrary, the longer the duration the greater the relative energetic contributions of both the glycolytic and oxidative energy systems (Fig. 3). Our results closely reflect the relative energy contributions when comparing the 15 s all-out cycle test reported by Yang et al. (2023)”. Noteworthy, the oxidative contribution time was not subtracted from the 15-s all-out sprint time, a crucial step for precise calculation of νLa_max_. This omission decreases the denominator of the formula, consequently inflating νLa_max_ when the time contributed by the oxidative system is separately deducted from the total exercise duration. This adjustment in the formula has the potential to alter the outcomes and interpretations of the study by Langley et al. for instance when concluding “[…] a 10-s test duration was optimal to elicit the highest VLamax in agreement with prior determination via mathematical modelling.”

As a final remark the formula of νLa_max_ was originated by Mader (1984). The reference (Heck et al. 2003) is incorrect for the νLa_max_ formula.

## References

[CR1] Driss T, Vandewalle H (2013) The measurement of maximal (anaerobic) power output on a cycle ergometer: a critical review. Biomed Res Int 2013:589361. 10.1155/2013/589361 24073413 10.1155/2013/589361 PMC3773392

[CR2] Heck H, Schulz H, Bartmus U (2003) Diagnostics of anaerobic power and capacity. Eur J Sport Sci 3(3):1–23

[CR3] Hopker J, Myers S, Jobson S, Bruce W, Passfield L (2010) Validity and reliability of the wattbike cycle ergometer. Int J Sports Med 31:731–736. 10.1055/s-0030-1261968 20665423 10.1055/s-0030-1261968

[CR4] Mader A (1984) Eine Theorie zur Berechnung der Dynamik und des steady state von Phosphorylierungszustand und Stoffwechselaktivität der Muskelzelle als Folge des Energiebedarfs. na

[CR5] Yang W-H, Park S-Y, Kim T, Jeon H-J, Heine O, Gehlert S (2023) A modified formula using energy system contributions to calculate pure maximal rate of lactate accumulation during a maximal sprint cycling test. Front Physiol. 10.3389/fphys.2023.1147321 37123252 10.3389/fphys.2023.1147321 PMC10133696

